# Screening, identification, metabolic pathway of di-n-butyl phthalate degrading *Priestia megaterium* P-7 isolated from long-term film mulched cotton field soil in Xinjiang

**DOI:** 10.3389/fmicb.2025.1538746

**Published:** 2025-04-30

**Authors:** Yuanyang Yi, Yuxian Wang, Wanqin Liu, Jing Zhu, Meiying Gu, Qiong Jia, Xue Li, Munire Mutalifu, Ling Jiang, Wei Zhang, Zhidong Zhang

**Affiliations:** ^1^College of Life Sciences, Xinjiang Normal University, Urumqi, China; ^2^College of Life Science and Technology, Xinjiang University, Urumqi, China; ^3^Xinjiang Key Laboratory of Special Environmental Microbiology, Institute of Applied Microbiology, Xinjiang Academy of Agricultural Science, Urumqi, China; ^4^College of Food Science and Pharmaceutical Science, Xinjiang Agricultural University, Urumqi, China; ^5^State Key Laboratory of Materials-Oriented Chemical Engineering, College of Food Science and Light Industry, Nanjing Tech University, Nanjing, China

**Keywords:** Di-n-butyl phthalate, biodegradation, whole-genome sequencing, metabolomics analysis, *Priestia megaterium* P-7

## Abstract

**Introduction:**

Di-n-butyl phthalate (DBP) is one of the most widely used phthalate esters (PAEs) and is considered an emerging global pollutant. It may pose a significant threat to ecosystem and human health due to its residual hazards and accumulation in the environment. Bacteria-driven PAE biodegradation is considered an economical and effective strategy for remediating such polluted environments.

**Methods:**

A DBP-degrading bacterium (P-7), was isolated from long-term film mulched cotton field soil. Its identity was confirmed via physiological, biochemical, and 16S rRNA gene analyses. The degradation conditions were optimized through single-factor experiments and response surface methodology (RSM).Furthermore, the whole-genome sequencing coupled with metabolomics was employed to elucidate metabolic mechanisms.

**Results:**

*Priestia megaterium* P-7 (*P. megaterium* P-7) achieved 100% DBP removal within 20 h under optimal conditions and exhibited broad substrate specificity for other PAEs. Genomic analysis identified key genes (*lip*, *aes*, *ybfF*, *estA*, and *yvaK*) encoding esterases/hydrolases that initiate DBP catabolism, converting it to phthalic acid (PA). Subsequent decarboxylation (*pdc*, *bsdCD*, *mdcACDH*, and *lysA*) and dioxygenase-mediated steps integrated PA into the TCA cycle. Metabolomics revealed three degradation pathways: decarboxylation (DBP → MBP → BB → BA→Catechol), hydrolysis (DBP → MBP → PA → PCA → Catechol) and direct β-oxidation (DBP → DEP → MEP → PA → Catechol).

**Conclusion:**

*P. megaterium* P-7 demonstrates exceptional degradation efficiency, substrate versatility, and environmental stress tolerance, making it a promising candidate for bioremediation of organic pollutants in contaminated soil.

## 1 Introduction

Phthalic acid esters (PAEs) are commonly incorporated into plastics as plasticizers to enhance the plasticity and flexibility. They are widely used in plastic films, pesticides, rubber, and various other plastic products (Chen et al., 2024). However, during the use of agricultural film, PAEs can migrate into the environment, leading to significant contamination of agricultural soils (Zhang et al., 2021). Research indicates that DBP is one of the major PAE contaminants in soil mulched with plastic film in cotton fields in Xinjiang (Huang et al., 2018). Its concentration is usually higher than other PAEs, reaching up to 57.7 mg/kg, far exceeding the soil DBP control standards set by the U.S. Environmental Protection Agency (USEPA) (Lü et al., 2018; Feng et al., 2024). As a typical environmental endocrine-disruptor, DBP has significant ecological toxicity effects. It not only causes delayed plant growth, reduced yield, and decreased quality, but also affects soil quality by reducing soil porosity, air permeability, and water permeability, thereby disrupting the function of soil microbial communities (Wang et al., 2021; Tao et al., 2023). In addition, even at extremely low concentrations, DBP has mutagenic, carcinogenic, and endocrine disrupting effects, causing toxicity to multiple organ systems including the immune system, nervous system, and reproductive system (Wang et al., 2023). Therefore, DBP has been listed as a priority pollutant by China Environmental Monitoring Terminal and USEPA (Liu et al., 2021). The accumulation of DBP in the long-term film mulched cotton field soils in Xinjiang has brought new challenges to agricultural production and ecological environment, and there is an urgent need to develop effective bioremediation technologies to address this issue.

In the remediation of DBP pollution, although physical and chemical methods are effective, they are costly and prone to secondary pollution, making them difficult to apply in the long-term (Ghosh and Sahu, 2022). On the contrary, due to the environmentally friendly and sustainable advantages of microbial degradation, it has gained widespread attention and is considered the most effective method for eliminating DBP in soil (Feng et al., 2021; Baker et al., 2021; Xu W. et al., 2022). At present, research on microorganisms that degrade PAEs mainly focuses on bacterial species. Extensive isolation and functional analysis have been conducted on PAEs degrading bacteria from environmental media such as activated sludge, sediment, soil, and plant tissue (Hu et al., 2021; Ren et al., 2023). These bacteria belong to the phyla such as Proteobacteria, Actinobacteria, Firmicutes, Chlorobi, and Deinococcus-Thermus, highlighting the broad bacterial diversity within the PAEs degrading microbial niche (Tao et al., 2023). Although many DBP degrading bacteria have been isolated in recent years, research on the degradation performance of different strains in actual soil environments is still limited. Considering the structural complexity of PAEs and the different degradation requirements in the environment, the degradation capacity and efficiency of soil microbial communities may vary significantly in different regions. Therefore, it is particularly necessary to explore the degradation potential of region-specific strains.

The soil microbial community in Xinjiang has formed a unique ecosystem under long-term film mulching cultivation (Zhang et al., 2016; Yu et al., 2023). This environment may encourage local microorganisms to develop stronger adaptability and degradation ability toward plastic film pollutants such as DBP. Previous studies have shown that certain microorganisms isolated from polluted environments in Xinjiang have the ability to degrade organic pollutants such as organic phosphorus and polycyclic aromatic hydrocarbons (Jin et al., 2020; Lü et al., 2023), providing a solid microbial resource foundation for screening DBP degrading bacteria. Further exploration of the microbial degradation potential in this region can reveal the ecological functions and adaptation mechanisms of these microbes in plastic film contaminated soil, and provide scientific evidence for the application of microbial remediation technology in film mulched cotton fields in Xinjiang. Therefore, the discovery and identification of microorganisms with DBP degradation ability in Xinjiang not only helps to restore the local agricultural ecological environment, but also provides new candidate strains for the bioremediation of PAEs pollutants.

The *Priestia* genus (formerly named *Bacillus*) has shown great potential in pollutant degradation (Liu et al., 2022). It is a Gram-positive, strict aerobic bacterium with strong environmental adaptability (Biedendieck et al., 2021). In recent years, studies have found that *Priestia* sp. can degrade various organic pollutants, including plastic additives and petroleum contaminants (Liu et al., 2022; Chakraborty et al., 2023). The unique environmental conditions and persistent plastic pollution in cotton fields in Xinjiang may promote the development of certain microbial strains capable of degrading PAEs, particularly those related to the highly adaptable *Priestia* sp. Previous studies have shown that *Priestia* sp. has strong degradation ability in harsh environments, enabling it to rapidly adapt and utilize organic pollutants as carbon sources (Sandhu et al., 2022; Wardhani et al., 2023). This degradation characteristic makes *Priestia* sp. an ideal candidate for investigating the potential of DBP degradation in cotton fields soils in Xinjiang. However, currently only a limited number of *Priestia* sp. have been reported to have the ability to degrade DBP. Therefore, studying the degradation pathway of *Priestia* sp. with efficient DBP degradation ability is of great significance for understanding the key steps and toxicological behavior of their metabolites, as well as inferring their biodegradation pathway.

This aim of this study is to screen and identify high DBP degrading strain to address DBP pollution in soil from long-term film mulched cotton fields in Xinjiang. This work screened a total of 8 DBP degrading bacterial strain from film mulched soil, among which the strain P-7 identified as *P. megaterium* exhibited higher degradation efficiency. Therefore, the DBP degradation capability of isolated *P. megaterium* P-7, along with its efficiency in degrading other PAEs, were evaluated. The genetic characteristics of the strain were explored, and optimal conditions for the biodegradation of DBP by *P. megaterium* P-7 were determined. Additionally, the DBP metabolic intermediates produced by *P. megaterium* P-7 were identified, and a potential metabolic pathway was proposed. These findings reveal the mechanism of *P. megaterium* P-7 in PAE degradation and provide a theoretical foundation for the bioremediation of PAE-contaminated soils, holding significant value for advancing farmland environmental management and pollution control.

## 2 Materials and methods

### 2.1 Materials

Dimethyl phthalate (DMP), diethyl phthalate (DEP), di-n-butyl phthalate (DBP), butyl benzyl phthalate (BBP), bis (2-ethylhexyl) phthalate (DEHP) (purity >98%) was purchased from Aladdin Chemistry Co., Ltd. (Shanghai, China). Chromatographic grade methanol and dichloromethane were purchased from Sigma (Germany). All organic solvents used were of chromatographic grade, and other chemical reagents were of analytical grade. The basic mineral salts medium (MSM) contained KH_2_PO_4_ (1.0 g/L), K_2_HPO_4_ (1.0 g/L), (NH_4_)_2_SO_4_ (1.0 g/L), MgSO_4_·7H_2_O (0.2 g/L), CaCl_2_ (0.02 g/L), and 1.0 mL trace element solution per liter of medium (39.9 mg/L MnSO_4_·H_2_O, 42.8 mg/L ZnSO_4_·H_2_O, and 34.7 mg/L (NH_4_)_6_Mo_7_O_24_·4H_2_O). The final pH of media was adjusted to 7.2 and then sterilized at 121°C in an autoclave for 20 min.

### 2.2 Enrichment and isolation of DBP degrading bacteria

The sampling location is in Wujiaqu City, Xinjiang, China (44°18′55″-44°40′00″N, 87°25′05″-87°36′05″E). This site is a cotton field mulched by agricultural plastic film for a long time. Five typical sampling points were selected, and the snake shaped sampling method was used to collect samples from the surface soil (0–20 cm). After removing stones, dead branches, leaves, and other impurities, the samples were thoroughly mixed, and 1 kg was retained by quartering. The samples were transported to the laboratory in waterproof paper bags and stored at 4°C. The enrichment culture method was used to isolate bacteria that could grow on DBP as the sole carbon source (Shariati et al., 2022). Briefly, 10 g of soil was placed into a flask containing 90 mL of sterile water, and shaked at 180 rpm for 30 min. After standing for 5 min, 2 mL of the suspension was added to 100 mL of MSM containing 50 mg/L of DBP. The obtained suspension was incubated at 30°C and 150 rpm for 4 days, followed by four series of subcultures, gradually increasing the DBP concentration (50, 100, 200, and 500 mg/L). Finally, 100 μL of the enriched culture from the fourth subculture flask containing 500 mg/L DBP was spread onto MSM agar medium supplemented with 100 mg/L DBP and incubated at 30°C for 72 h. Subsequently, individual bacterial colonies were inoculated onto MSM agar plates containing 100 mg/L of DBP for further purification. The colonies grown on MSM agar plates were identified as potential DBP degrading strains.

### 2.3 Identification of DBP degrading bacteria

The strain with high DBP degrading ability was selected for further identification. The morphology observation was carried out by optical microscope and scanning electron microscopy (SEM). The genomic DNA of the isolated strain was extracted using a DNA extraction kit (Omega BioTek, United States). The bacterial 16S rRNA gene was amplified using universal PCR primers 27F (5′-AGAGTTTGATCCTGGCTCAG-3′) and 1492R (5′-GGTTACCT TGTTACGACTT-3′). The PCR products were purified on 1% agarose gel, and sequenced by Sangon Biotech Co., Ltd. (Shanghai, China). The sequence of the isolate was compared with known sequences in the NCBI GenBank database to search for relevant reference strains with high sequence similarity. A phylogenetic tree was constructed using MEGA software (version 11.0).

### 2.4 Substrate utilization test of isolated strain

The substrate utilization efficiency for PAEs by isolated strain was determined by testing the growth ability in liquid MSM supplemented with one of the following substrates: DMP, DEP, DBP, BBP, and DEHP. The detail experimental procedure was as follows. The purified strains were inoculated into R2A medium and cultured for 24 h at 30°C and 150 rpm. Subsequently, the cultured bacterial solution (2%, v/v) was inoculated into fresh MSM containing different PAEs (100 mg/L) as sole source of carbon and energy. After 3 days of cultivation, residual PAEs were extracted and purified from the culture medium according to the method reported in previous literature (Feng N. et al., 2018). The extracts were analyzed using gas chromatography–mass spectrometry (GC–MS, Aglient 7893-5975) with a DB-5 MS capillary column (length 30 m, i.d. 0.25 mm, film 0.25 μm, Agilent). The carrier gas was helium (99.999% purity) with a constant flow of 1 mL/min. The carrier gas was helium (99.999% purity) with a constant flow of 1 mL/min. The temperatures for the transfer line and ion source were 280°C and 230°C, respectively. Analyte ionization was performed using electron ionization (70 eV), and signal acquisition was performed in selected ion-monitoring (SIM) mode. The PAEs recoveries in samples ranged from 82.40 to 103.50%. The detection limit of PAEs in samples was 0.05–0.15 μg/L. The degradation rate of the strain was calculated according to the following formula: Degradation rate (%) = (1-*C*/*C_0_*) × 100%, where *C* and *C_0_* represent the concentration of PAEs in inoculated and non-inoculated media, respectively. All experiments were repeated three times.

### 2.5 Optimization of DBP degrading conditions

The optimal parameters for the maximal degradation rate of DBP by strain P-7 were initially determined through single-factor experimental design. These parameters included pH (5 ~ 9), inoculation amount (1 ~ 5%), metal ions (Fe^2+^, Ca^2+^, Mn^2+^, Mg^2+^, and Cu^2+^), carbon sources (dextrin, corn, starch, sucrose, glucose, xylose), and nitrogen sources (yeast extract, tryptone, urea, ammonium sulfate, beef extract). Subsequently, based on the single-factor experimental results, principal component analysis (PCA) and response surface methodology (RSM) were used to optimize the culture conditions for efficient DBP degradation by strain P-7, which was used to select the three key factors involved in the DBP degradation. By comprehensive score of above five growth factors (Supplementary Table S1), three important factors (metal ions, carbon source, and nitrogen source) were identified from PCA results as the independent variables for RSM analysis. Based on the Box–Behnken design, the range of independent-variables and levels are shown in Supplementary Table S2. The RSM was further applied to optimize the degradation conditions (Alghamdi et al., 2023), resulting in 15 experiments with each independent variable at three different levels (−1, 0, and +1) using Box–Behnken (Table 1). A Box–Behnken matrix and the response of dependent variable for DBP degradation were generated using Design Expert. All experiments were conducted in 100 mL Erlenmeyer flasks containing 20 mL of trypticase soy broth (TSB) medium and 100 mg/mL of DBP. After incubation for 8 h, the residual DBP in medium was measured to calculate the degradation rate. A group without strain P-7 was used as control (CK), and all treatments were performed in triplicates.

**Table 1:** Box–Behnken design matrix and the response of dependent variable for DBP degradation.

Run	Code levels of independent variables			Response
	X_1_	X_2_	X_3_	Y (%)
1	−1	−1	0	68.02 ± 2.1c
2	1	−1	0	69.4 ± 2.6b
3	−1	1	0	67.5 ± 1.6d
4	1	1	0	65.3 ± 2.3b
5	−1	0	−1	68.50 ± 2.6ab
6	1	0	−1	73.35 ± 2.5bc
7	−1	0	1	72.34 ± 2.8c
8	1	0	1	60.4 ± 2.2b
9	0	−1	−1	69.44 ± 3.4b
10	0	1	−1	70.5 ± 2.7c
11	0	−1	1	65.11 ± 1.8d
12	0	1	1	69.38 ± 1.4c
13	0	0	0	75.4 ± 2.8bc
14	0	0	0	79.11 ± 1.8d
15	0	0	0	77.91 ± 2.2b

X_1_ refers to beef extract; X_2_ refers to sucrose; X_3_ refers to Fe^2+^; Y refers to the degradation rate. The data are means of three replicates with standard deviation. Data followed by the same letters in the same column are not significantly different at *p* = 0.05 level according to the Duncan’s multiple range test.

The obtained data were finally analyzed using the response surface regression procedure to fit the following second-order polynomial equation (Du et al., 2024):


$$Y=\beta_{0}+\sum \beta_{i}X_{i}+\sum \beta_{ij}X_{i}X_{j}+\sum \beta_{ii}X_{i}^{2}$$

Where *Y* is the response variable (DBP degradation), *X_i_* and *X_j_* are the independent variables (metal ions, carbon source, and nitrogen source), *β*_0_ is the constant coefficient, *β_i_* is the line coefficient, *β_ij_* is the interaction coefficient, and *β_ii_* is the quadratic coefficient. ANOVA and *F*-value were performed to evaluate the statistical significance and efficiency of the model. The multiple determination coefficient (R^2^) was calculated to indicate the suitability of model. To demonstrate the individual and interactive effects of the independent variables on DBP degradation, three-dimensional response surface plots and contour plots were constructed to intuitively predict the impact of three factors on degradation interactions.

### 2.6 Whole-genome sequencing and annotation of DBP degrading bacteria

The strain P-7 cells were harvested for further DNA extraction using Wizard^®^ Genomic DNA Purification Kit (Promega, United States). The size, quantity and quality of purified DNA were checked by TBS-380 fluorometer (Turner BioSystem Inc., United States). The whole genome was sequenced using Illumina HiSeq platforms and PacBio RS II Single Molecule Real Time (SMRT) system at Shanghai Majorbio Bio-pharm Technology CO., Ltd. (Majorbio, China). The raw reads obtained were trimmed into clean reads, and then assembled into a contig by Unicycle v0.4.8. The bioinformatic analysis was performed using Majorbio Cloud Platform.^1^ The protein-coding sequences (CDSs) were predicted using Glimmer.^2^ The following databases were used for gene functional annotation: Gene Ontology (GO), Kyoto Encyclopedia of Genes and Genomes (KEGG), Cluster of Orthologous Groups (COG), Swiss-Prot, and non-redundant protein.

### 2.7 Analysis of DBP degrading intermediates

To analyze the metabolites involved in DBP degradation, bacterial cells were inoculated at a 2% inoculation volume into MSM liquid medium containing 100 mg/L DBP. Then, all cultures were incubated at 30°C with shaking at 180 rpm for 24 h. Samples were collected at 0, 6, 8, and 18 h. Metabolic intermediates were extracted using an equal volume of n-hexane, dried under nitrogen gas (purity > 99.99%), and redissolved in methanol. The solution was filtered through a 0.22 μm membrane, and the filtrate was transferred into 2 mL glass vials for identification. Finally, the metabolic intermediates of DBP were determined by Panomix CO., Ltd. (Suzhou, China) using ultra-high performance liquid chromatography-mass spectrometry (UHPLC–MS/MS) (Thermo Fisher Scientific, United States).

### 2.8 Statistical analysis

All statistical analysis were conducted using SPSS 22.0 software. Statistical significance was set at *p* < 0.05. The data were fitted and plotted using Origin Pro. 2021. Other sequencing results were directly generated from the Majorbio Cloud Platform (see Footnote 1). All experiments were carried out in triplicate.

## 3 Results

### 3.1 Isolation and identification of DBP degrading bacteria from film mulched soil

**Figure 1:**
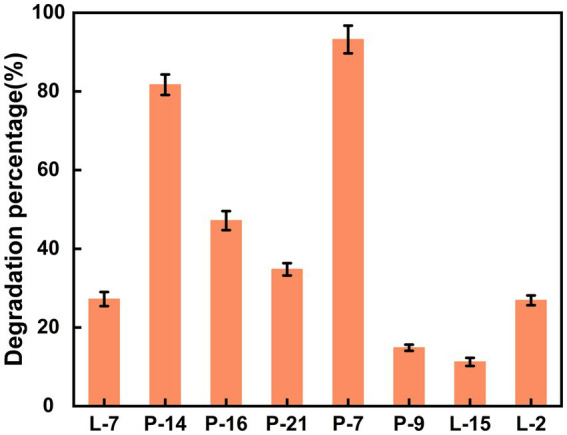
The biodegradation efficiency of DBP by 8 isolated bacteria.

Using DBP as the sole carbon and energy source, 8 bacterial strains capable of utilizing DBP were isolated from film mulched cotton field soil through enrichment and adaptation (Supplementary Table S3). The isolated strains, designated as L-2, L-7, L-15, P-7, P-9, P-14, P-16, and P-21, showed varied efficiencies in DBP biodegradation (Figure 1). Overall, all 8 strains exhibited the ability to degrade DBP, though with significant differences in degradation efficiencies. Strains L-2, L-7, and P-21 demonstrated degradation rates of less than 40% within 3 days, while strains P-9 and L − 15 showed degradation rates of less than 20%, indicating relatively low degradation capability. In contrast, strains P-7 and P-14 achieved higher degradation efficiency, with strain P-7 performing best, reaching over 93%. These results suggest that strain P-7 holds great potential as an efficient bacterial strain for DBP degradation in the environment. Therefore, it was selected as a candidate for further cultivation optimization and DBP biodegradation pathway study.

**Figure 2:**
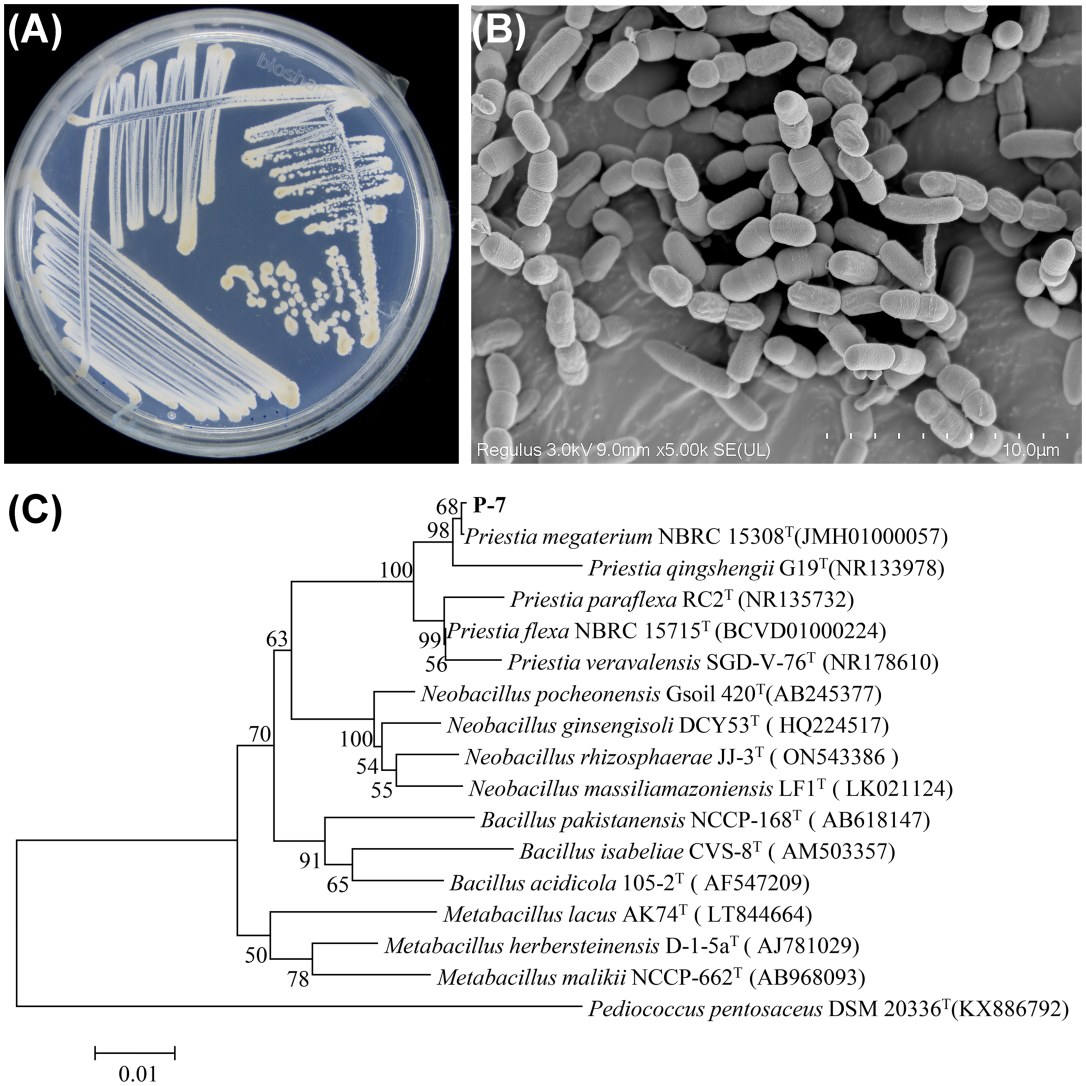
**(A)** Colony characteristics of *P. megaterium* P-7 on an R2A agar plate. **(B)** SEM morphology of *P. megaterium* P-7. **(C)** Phylogenetic tree analysis based on the 16S rRNA gene sequence of *P. megaterium* P-7.

Next, the isolated strain P-7 was identified according to the biochemical identification, morphological observation, and 16S rRNA gene sequencing. As shown in Supplementary Table S4, the physiological and biochemical assays indicated that strain P-7 was a Gram-positive strain with strong tolerance to salt, temperature, alkali, and phenol. Additionally, it was capable of producing catalase and protease, which would help to catalyze DBP and other organic pollutants. After 24 h culturation on R2A agar medium, the colonies of strain P-7 were faint yellow, with a smooth, moist surface, regular edges, and an opaque appearance (Figure 2A). SEM observation revealed that strain P-7 cells were short and rod-shaped with a width of 1.5–2.0 μm and length of 4.2–7.8 μm (Figure 2B). Further comparison of the 16S rRNA gene sequence of strain P-7 with NCBI database and phylogenetic tree construction showed that strain P-7 belonged to the genus *Priestia* and clustered with *Priestia megaterium* NBRC 15308 (JMH01000057) with a homology of over 99% (Figure 2C). In conclusion, strain P-7 was ultimately identified as *Priestia megaterium* and designated as *P. megaterium* P-7.

### 3.2 Optimization of DBP degrading conditions

**Figure 3:**
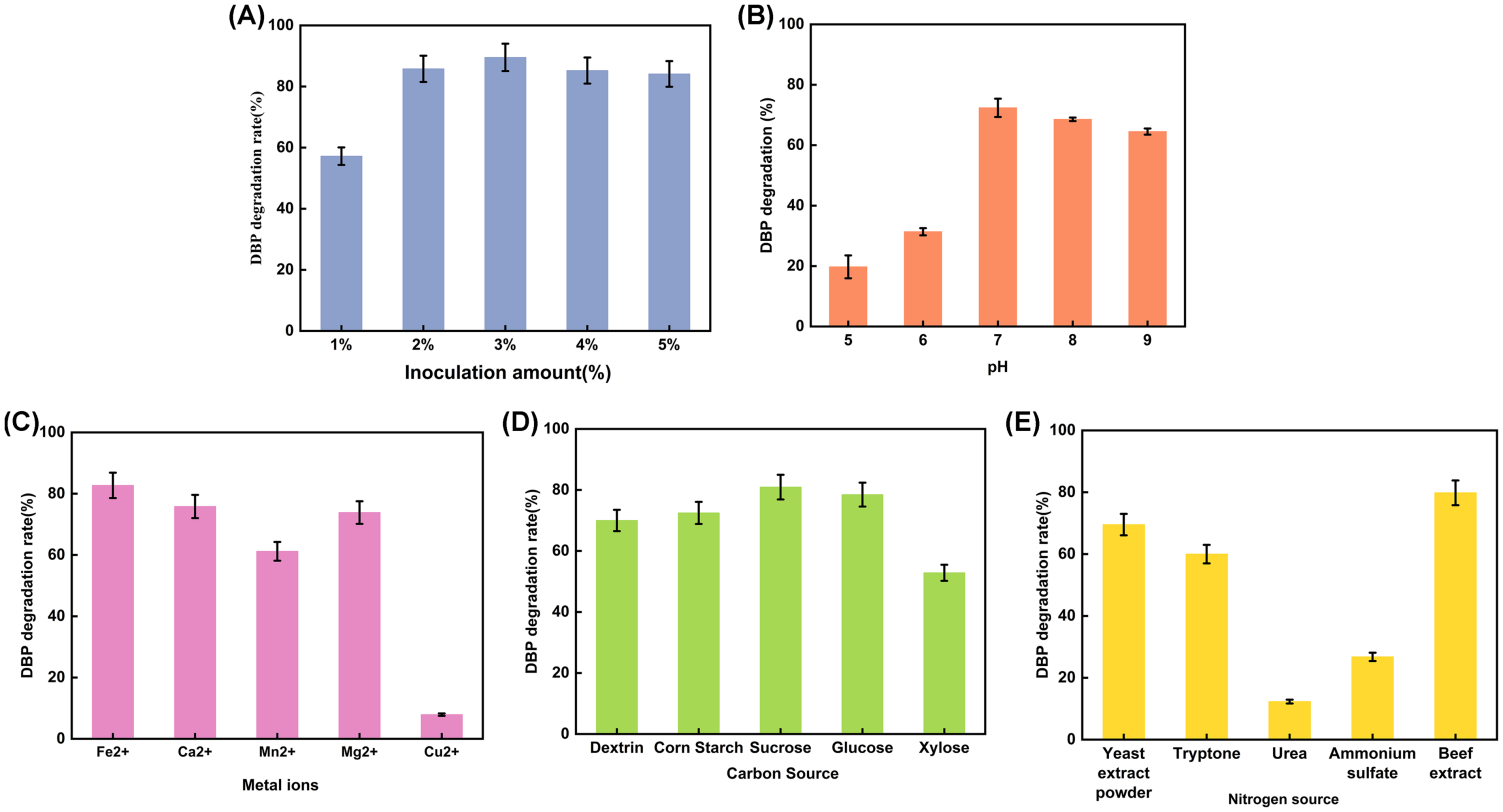
DBP degradation rate of *P. megaterium* P-7 under different environmental factors: **(A)** inoculation amount, **(B)** pH, **(C)** metal ions, **(D)** carbon source, and **(E)** nitrogen source.

To obtain the optimal conditions for *P. megaterium* P-7 to degrade DBP, single-factor experiment was conducted to explore the effects of five different growth factors (pH, inoculation amount, metal ions, carbon and nitrogen source) on DBP degradation. As shown in Figure 3A, a low inoculum amount (1%) of *P. megaterium* P-7 suspension was able to achieve a DBP degradation efficiency of nearly 60%. Further increasing the inoculum amount, the degradation rate of DBP remained stable at over 80%, indicating the enormous potential of *P. megaterium* P-7 for DBP degradation. In addition, *P. megaterium* P-7 was sensitive to pH, especially under acidic condition (Figure 3B). The DBP degradation rate increased rapidly when the pH was adjusted to neutral (pH 7.0) and weakly alkaline conditions (pH 9.0). Notably, it was observed that the significant enhancement in DBP degradation was effectively supported at low concentrations of metal ions except for Cu^2+^, in the following order: Fe^2+^ > Mg^2+^ > Ca^2+^ > Mn^2+^ > Cu^2+^ (Figure 3C), which was mainly achieved by affecting enzyme activity. Moreover, the supplementation of carbon and nitrogen sources also significantly increased the DBP degradation efficiency of *P. megaterium* P-7. Among the tested carbon sources, sucrose, glucose, corn starch, dextrin, and xylose proved to be suitable options, demonstrating a broad adaptability (Figure 3D). In contrast, the effective nitrogen sources were relatively limited, mainly including beef extract, yeast extract, and tryptone (Figure 3E). Based on the above results, we determined that the optimal metal ions and carbon/nitrogen sources for DBP degradation by *P. megaterium* P-7 were Fe^2+^, sucrose and beef extract.

To optimize the conditions for DBP biodegradation, single-factor experiments were first conducted, followed by PCA to identify the key factors influencing the degradation rate. The relationship between the DBP degradation rate and the three main factors was further explored using RSM design matrix, along with a polynomial regression model, to determine the optimal combination of variables. As shown in Table 2, the ANOVA for the quadratic response surface model fit (*R*^2^ = 0.9246) indicates that the model can explain more than 92% of the experimental and predicted data. Additionally, the adjusted *R*^2^ (0.7888) is also acceptable, confirming the model’s accuracy and reliability. According to the results of the *F*-test and *t*-test, the factors affecting DBP degradation rate are ranked as follows: Fe^2+^ > beef extract > sucrose. Only the linear term (AC) and the quadratic terms (A^2^ and B^2^) significantly affect DBP biodegradation (*p* < 0.05). Therefore, the predictive equation for DBP degradation rate (%) with significant model terms is as follows:


$$\begin{array}{l} Y=-201.486+29.42A+806.57B+1147.52C-9.22\mathrm{AB}\\ \;-55.87\mathrm{AC}+427.85BC-1.23A^{2}-1987.85B^{2}-2781.99C^{2} \end{array}$$

**Table 2:** ANOVA of the regression model for DBP degradation by *P. megaterium* P-7.

Source	Sum of squares	Degrees of freedom	Mean square	*F-*value	*p*-value
Model	316.43	9	35.16	6.81	0.024^*^
A	7.7	1	7.7	1.49	0.2764
B	0.0673	1	0.0673	0.013	0.9136
C	26.4	1	26.4	5.11	0.0733
AB	3.4	1	3.4	0.6587	0.4539
AC	70.23	1	70.23	13.6	0.0142^*^
BC	2.57	1	2.57	0.4985	0.5117
A^2^	89.41	1	89.41	17.31	0.0088^**^
B^2^	91.19	1	91.19	17.66	0.0085^**^
C^2^	56.51	1	56.51	10.94	0.0213^*^
Residual	25.82	5	5.16		
Lack of fit	18.87	3	6.29	1.81	0.3755
Pure error	6.96	2	3.48		
Total	342.25	14			

*R2 = 0.9246 R2Adj = 0.7888.*

A: beef extract; B: sucrose; C: Fe^2+^. The symbol (*) means the model terms are significant (*p* < 0.05).

Where Y is the DBP degradation rate (%), A is the beef extract content (g/L), B is the sucrose content (g/L), and C is the Fe^2+^ content (g/L).

**Figure 4:**
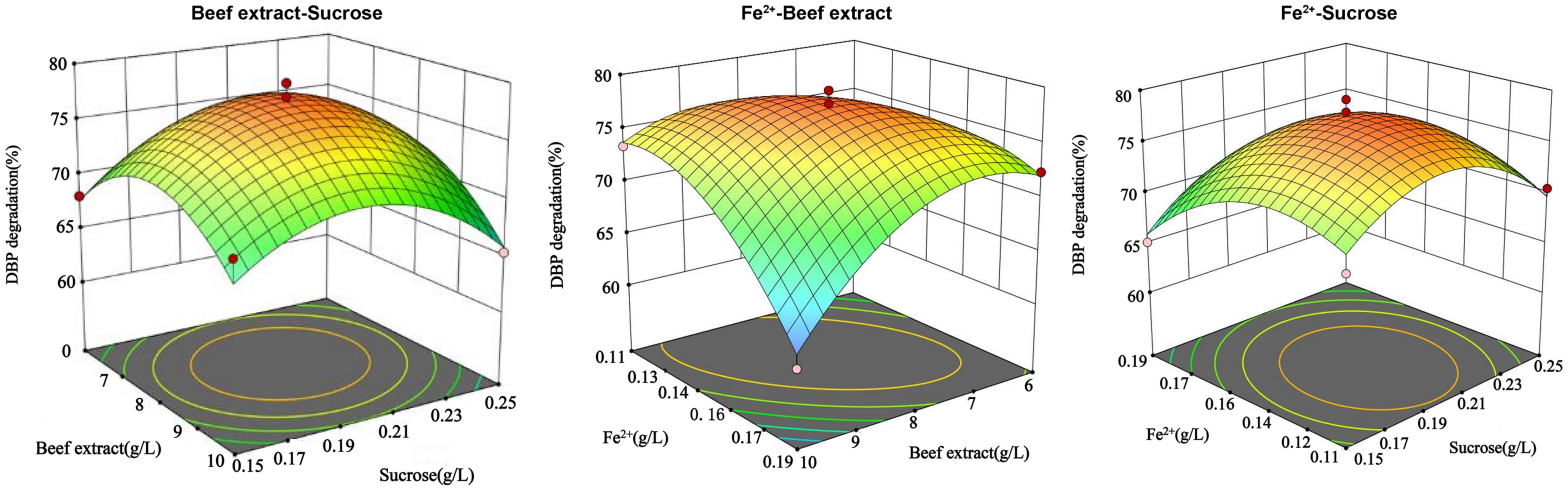
Three-dimensional response surface plots of three response factors (beef extract, Fe^2+^, and sucrose) for DBP degradation rate by *P. megaterium* P-7.

The experimental results were analyzed using Design-Expert 13.0 software, and the response surface was plotted. The interaction between the factors can be observed in Figure 4. When Fe^2+^ is at the zero level, the optimal concentrations of beef extract and sucrose are 6.44 g/L and 0.16 g/L, respectively. When sucrose is at the zero level, the optimal concentrations of beef extract and Fe^2+^ are 7.06 g/L and 0.18 g/L, respectively. The optimal conditions obtained from the mathematical model are a beef extract concentration of 9.92 g/L, sucrose concentration of 0.19 g/L, and Fe^2+^ concentration of 0.12 g/L. Under these conditions, the model predicts that *P. megaterium* P-7 will achieve the highest DBP degradation rate of 74.31%. A verification experiment was carried out under the optimal conditions, and the actual DBP degradation rate was found to be 78.5%. The difference between the predicted and experimental values is only 4.19%, confirming the validity and effectiveness of the optimized model.

Under these optimal degradation conditions, the relationship between the growth and DBP degradation of *P. megaterium* P-7 was achieved. As shown in Supplementary Figure S1, *P. megaterium* P-7 was able to rapidly degrade DBP and utilized it as a growth substrate, with DBP degradation positively correlated with bacterial growth. Moreover, the growth of *P. megaterium* P-7 exhibited no significant lag phase in the presence of DBP, but quickly entered the exponential growth phase, with 0–12 h being the exponential phase. During this time, the degradation rate of DBP increased rapidly and synchronously. As the growth of *P. megaterium* P-7 reached a stationary phase (12–20 h), the degradation curve also tended to stabilize. Ultimately, DBP in the culture medium was almost undetectable and the strain began to enter a decline phase, indicating the complete consumption of the growth substrate. The comparison of DBP biodegradation among different strains further demonstrates the superior degradation capability of *P. megaterium* P-7 (Supplementary Table S5), providing important theoretical support and potential applications for the bioremediation of DBP-induced soil pollution.

### 3.3 Substrate utilization of *Priestia megaterium* P-7

To evaluate substrate utilization, *P. megaterium* P-7 was cultured in liquid medium supplemented with 100 mg/L of each PAE substrates, including DMP, DEP, DBP, BBP, and DEHP. As shown in Supplementary Figure S2, the results indicated that *P. megaterium* P-7 was capable of utilizing all five PAEs as sole carbon sources, demonstrating the effective degradation ability. Notably, the strain *P. megaterium* P-7 exhibited a significantly higher degradation rate (over 60%) for PAEs with shorter side chains (DMP, DEP, DBP, and BBP) compare to DEHP, which has longer side chains. These findings highlight the strong potential of *P. megaterium* P-7 for efficient PAEs degradation and its promise as a candidate for bioremediation of PAE-contaminated environments.

### 3.4 Whole-genome analysis of *Priestia megaterium* P-7

**Figure 5:**
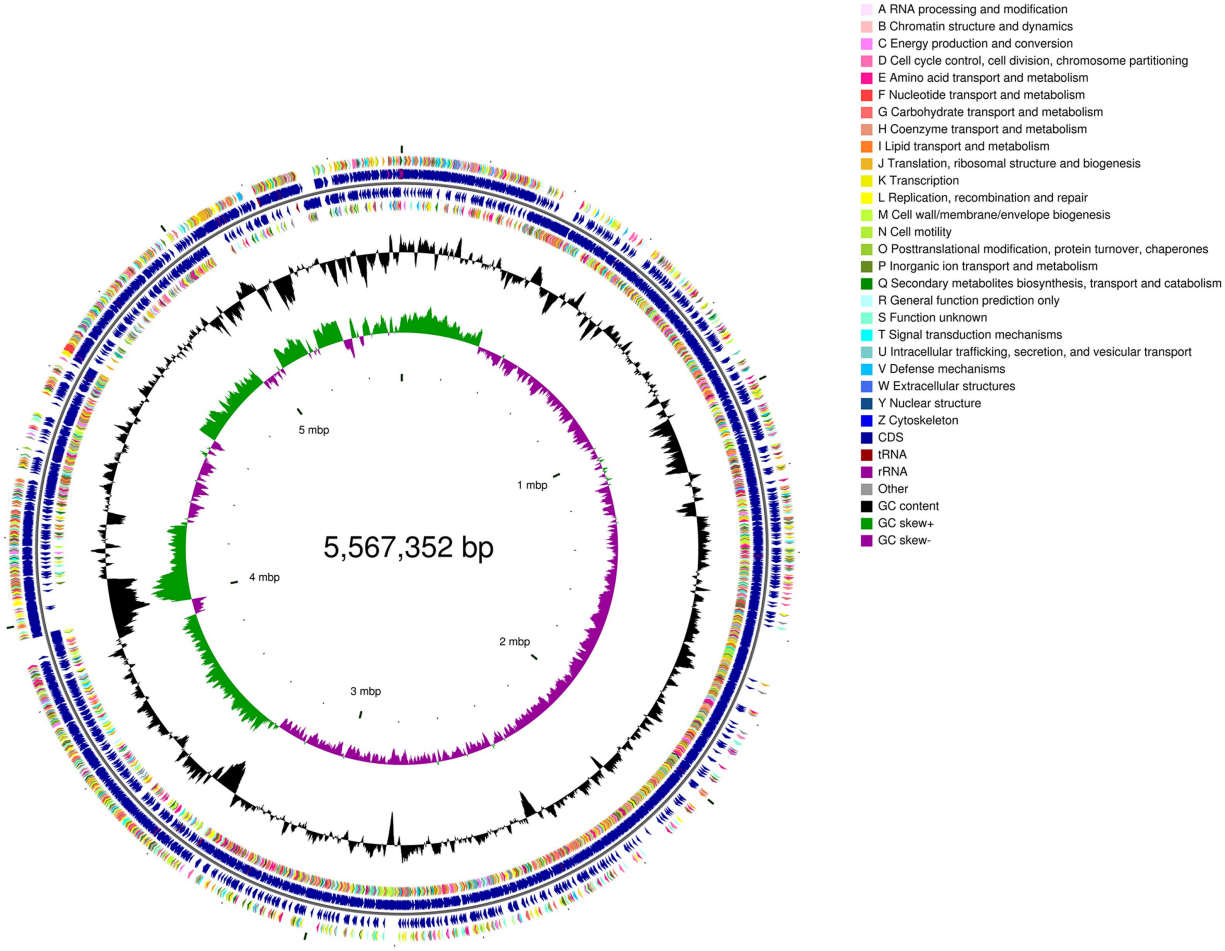
Circular genome map of *P. megaterium* P-7 based on genome sequence and annotation. From outside to inside, the first and fourth circles represent the CDS on forward and reverse chains, the second and third circles represent CDS, tRNA, and rRNA on forward and reverse chains, the fifth circle is the GC content, and the sixth circle represents the GC Skew value.

**Figure 6:**
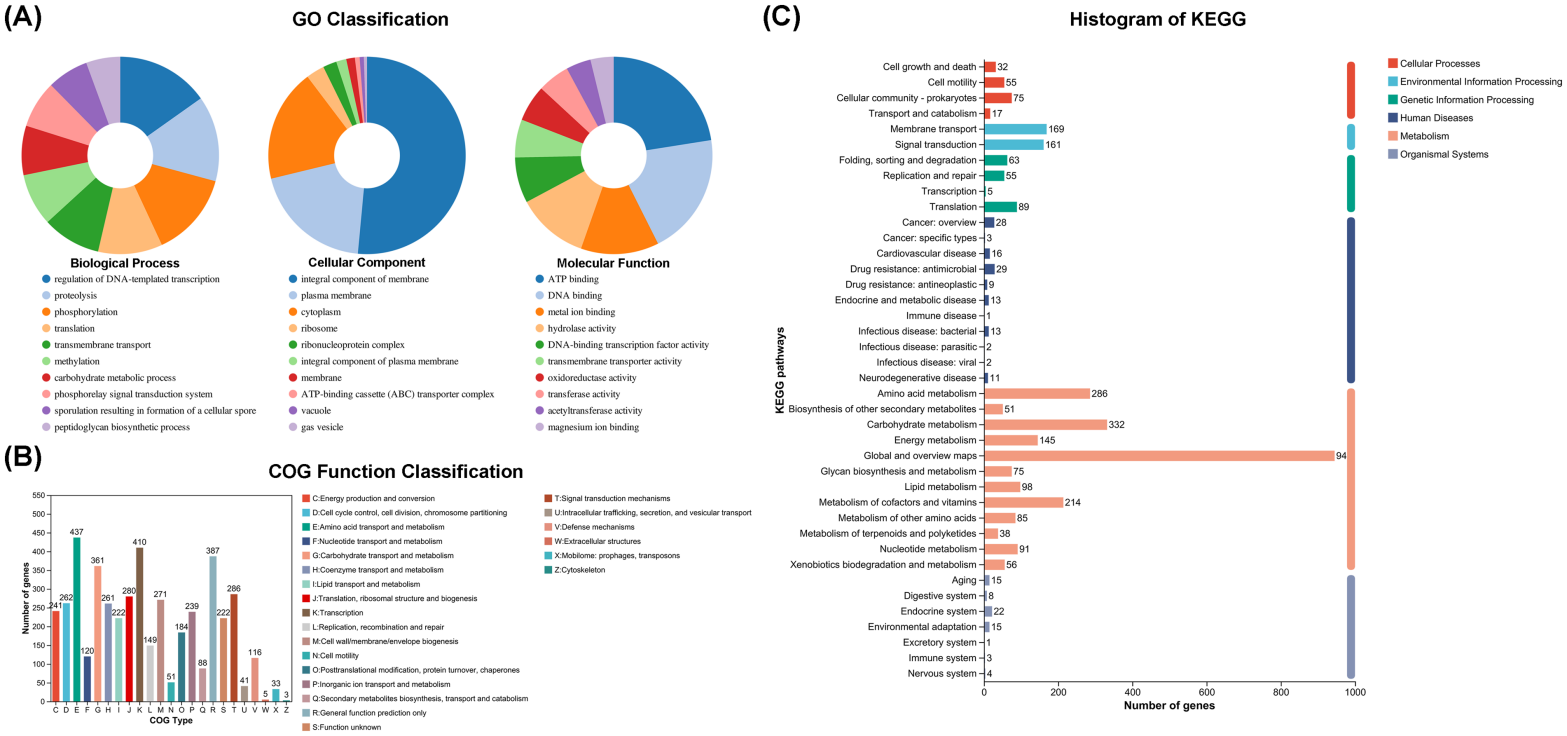
Function annotation of *P. megaterium* P-7. **(A)** GO classification annotation, **(B)** COG classification annotation, and **(C)** KEGG pathway classification histogram.

To further decipher the genetic information of *P. megaterium* P-7 and explore its functional genes, whole-genome sequencing and genomic analysis of this strain were conducted. The genomic characteristics of *P. megaterium* P-7 were summarized in Supplementary Table S6 and Figure 5. The genome of *P. megaterium* P-7 consisted of a single circular chromosome and was 5,567,352 bp in length with a GC content of 37.61%, of which 4,660,332 bp encodes genes. It contained a total of 5,653 predicted protein-coding sequences (CDSs), accounting for 83.71% of the total genome length. At the same time, there were 103 tRNA genes and 17 rRNA genes (1 of 16S rRNA, 1 of 23S rRNA, and 15 of 5S rRNA). These protein-coding genes were subjected to functional annotation using multiple databases, including GO, NR, COG, KEGG, Swiss-Prot, and Pfam databases. GO functional annotation results in Figure 6A showed that a total of 261 CDSs were mainly related to “regulation of DNA-templated transcription” (1.59%), “proteolysis” (1.47%) and “phosphorylation” (1.46%) in terms of biological processes. For cell composition, the CDSs were mainly related to “integral component of membrane” (17.13%), plasma membrane (6.57%), and cytoplasm (6.15%), while the genes were mainly related to ATP binding, DNA binding based on molecular function. Based on COG functional annotation, 4,669 CDSs were classified into 23 COG function classes, with the three most abundant categories being amino acid transport and metabolism, transcription, and general function prediction only (Figure 6B). According to KEGG pathway annotation, it was found that a total of 3,332 CDSs could be assigned to 40 metabolic pathways, which were involved in 6 biological pathways: cellular processes, environmental information processing, genetic information processing, human diseases, metabolism, organismal systems (Figure 6C). Among these genes, the majority were related to metabolic pathways, with 286 genes annotated for amino acid metabolism, 332 genes annotated for carbohydrate metabolism, and 214 genes annotated for metabolism of cofactors and vitamins. In addition, 56 genes associated with 14 pathways of xenobiotics biodegradation and metabolism was found. Especially, 33, 7, 8, and 9 genes were annotated to benzoate degradation, dioxin degradation, aminobenzoate degradation and xylene degradation, which exhibited the potential to degrade and metabolize organic pollutants in *P. megaterium* P-7.

### 3.5 Genome annotation related to DBP degrading enzymes

Based on the genome annotation of *P. megaterium* P-7, multiple genes and gene clusters potentially involved in the degradation of DBP were identified, including those encoding esterases, hydrolases, decarboxylases, and dioxygenases (Table 3). The positions of genes and gene clusters implicated in DBP degradation were delineated within the chromosomal genome (Supplementary Figure S3A), and their structural organization was characterized (Supplementary Figure S3B). Additionally, five promoter sequences were predicted among the 28 key genes (Supplementary Table S7). Key genes widely associated with ester bond hydrolysis and transesterification were annotated, such as *lip* (triacylglycerol lipase), *aes* (acetyl esterase), *ybfF* (esterase), *estA* (putative tributyrin esterase), and *yvaK* (carboxylesterase). These enzymes played a critical role in converting DBP to MBP and PA (Nahurira et al., 2019; Yoo et al., 2020; Wang et al., 2024). Subsequently, the genes encoding decarboxylases and dioxygenases facilitated the conversion of above intermediates into protocatechuate (PCA), which was a key intermediate in the aerobic degradation pathway of aromatic compounds (Geiger et al., 2019). According to the known metabolic pathways of PAEs, it was found that *pdc* (phenolic acid decarboxylase), *bsdCD* (4-hydroxybenzoate decarboxylase subunit), *mdcACDH* (malonate decarboxylase alpha subunit), and *lysA* (diaminopimelate decarboxylase) genes could catalyze the decarboxylation of MBP and PA to produce benzoic acid. Further steps are accomplished by the hydroxylation of the aromatic ring by dioxygenases to form the common intermediates such as catechol and PCA. Although only 4 genes encoding dioxygenases (K07104, K08967, K00452, K00453) were annotated using KEGG analysis, no previously reported phthalate dioxygenase genes (*pht* gene clusters) were identified in this study (Feng L. et al., 2018). However, Swiss-Prot annotation revealed two dioxygenase genes (*gene1285* and *gene1474*) involved in aromatic ring hydroxylation. Meanwhile, the ring of aromatic compounds was further opened by ring-cleaving dioxygenase (gene0497, gene3652, and gene5204) and catechol 2,3-dioxygenase (*catE*) to produce muconate derivative. These intermediates subsequently enter the catabolic pathway via the *β-ketoadipate oxidized to acetyl-CoA* and are ultimately entered the tricarboxylic acid (TCA) cycle, leading to the production of H₂O and CO₂ (Al-Khalid and Ei-Naas, 2012).

**Table 3:** Key genes for DBP degradation in *P. megaterium* P-7 based on KEGG and Swiss-Prot annotation.

Gene ID	KO ID	Gene name	Function description (KEGG/Swiss-Prot)
Esterases/hydrolases			
gene0529	K01066	*aes*	Acetyl esterase [EC:3.1.1.-]
gene1165	K01175	*ybfF*	Esterase [EC:3.1.-.-]
gene4373/ gene4638	K03928	*yvaK*	Carboxylesterase [EC:3.1.1.1]
gene4831	K03930	*estA*	Putative tributyrin esterase [EC:3.1.1.-]
gene3461	K01617	*dmpH*	2-hydroxyhexa-2,4-dienoate hydratase
Decarboxylases			
gene0156	K21759	*bsdD*	Vanillate/4-hydroxybenzoate decarboxylase subunit D
gene0457/gene1920	K01586	*lysA*	Diaminopimelate decarboxylase [EC:4.1.1.20]
gene1069/gene3430/gene3448/gene4613	K01607	*pcaC*	4-carboxymuconolactone decarboxylase
gene3189	K13932	*mdcD*	Malonate decarboxylase beta subunit [EC:4.1.1.87]
gene3190	K13931	*mdcC*	Malonate decarboxylase delta subunit
gene3192	K13929	*mdcA*	Malonate decarboxylase alpha subunit [EC:2.3.1.187]
gene3193	K13935	*mdcH*	Malonate decarboxylase epsilon subunit [EC:2.3.1.39]
gene4994	K13727	*pdc*	Phenolic acid decarboxylase [EC:4.1.1.-]
gene0155	K01612	*bsdC*	UbiD family decarboxylase, phenolic acid decarboxylase
Dioxygenases			
gene1517	K07104	*catE*	Catechol 2,3-dioxygenase [EC:1.13.11.2]
gene2754	K08967	*mtnD*	1,2-dihydroxy-3-keto-5-methylthiopentene dioxygenase
gene3454	K00452	*–*	3-hydroxyanthranilate 3,4-dioxygenase [EC:1.13.11.6]
gene3464	K00453	*kynA*	Tryptophan 2,3-dioxygenase [EC:1.13.11.11]
gene0497/gene3652/gene5204	K15975	*–*	Ring-cleaving dioxygenase
gene1285/gene1474	–	*–*	Aromatic ring-hydroxylating dioxygenase subunit

### 3.6 DBP biodegradation pathway of *Priestia megaterium* P-7

**Figure 7:**
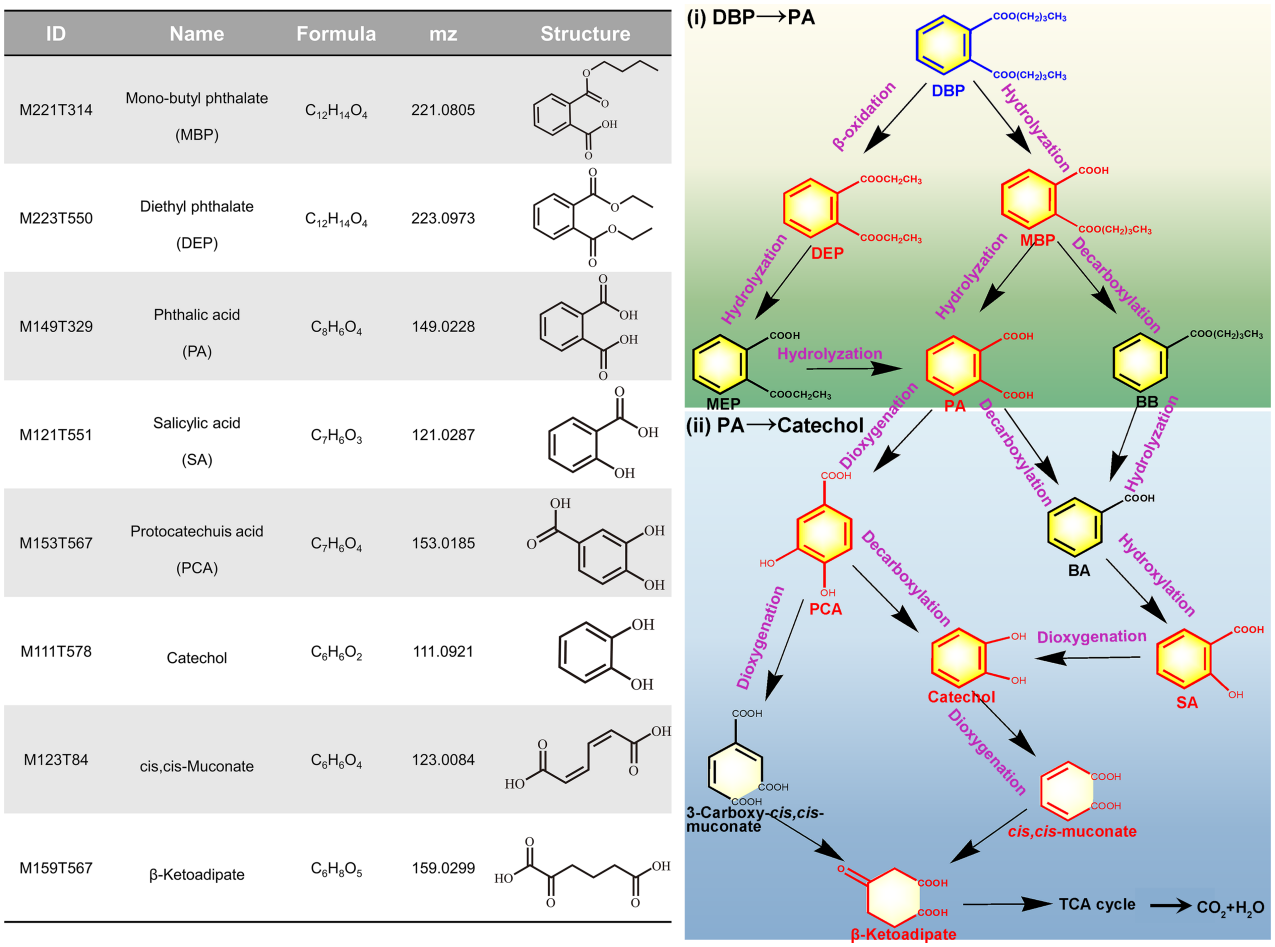
Metabolites of DBP degradation identified in *P. megaterium* P-7 and the proposed metabolic pathways.

To further elucidate the metabolic mechanism of DBP in *P. megaterium* P-7, the metabolic intermediates were preliminary measured based on UHPLC–MS/MS (Supplementary Figure S4). The metabolomics results indicated that a total of 2,457 metabolites were determined, among which 8 key intermediate products were closely related to DBP degradation (Figure 7; Supplementary Table S8). According to the identification of metabolic intermediates, an alkyl chain (-C_4_H_9_) in DBP was hydrolyzed by esterases to form the primary metabolites MBP, followed by the hydrolysis of another alky chain to form PA. Further, PA was converted to catechol via PCA under the reaction of dioxygenases. Finally, the benzene ring of catechol was opened to form cis, cis-muconate, which was further used for cell growth through the β-ketoadipate pathway (Chen et al., 2021). It has been reported that benzoic acid metabolic pathway is a key pathway for bacterial degradation of DBP, which is generated through the hydrolysis of MBP and the decarboxylation of PA (Feng et al., 2024). However, we did not identify benzoic acid among the metabolites, but instead identified its derivatives, such as salicylic acid and 4-hydroxybenzoic acid. Therefore, we hypothesized another metabolic pathway in which the primary metabolite MBP was transformed to BB under the catalysis of decarboxylase, followed by ester hydrolysis to form BA. Additionally, diethyl phthalate (DEP) was also detected and identified in the metabolites of the fermentation broth, which was generally formed by β-oxidation of DBP (Feng et al., 2024; Ren et al., 2024). This observation indicated that DBP degradation occurred via β-oxidation, resulting in the formation of DEP, which was further transformed to PA through de-esterification and subsequently entered PA metabolic pathway. Based on whole genome analysis and identification of metabolites, we ultimately proposed the detailed metabolic pathway of DBP in *P. megaterium* P-7, which was presented in Figure 7.

## 4 Discussion

PAEs are a widely used class of persistent plasticizers that are difficult to degrade, remaining in the environment for long periods and exhibiting bio-accumulative potential (Liu et al., 2024). Their pollution, particularly in soil and water environments, has raised widespread concerns. PAEs are frequently detected in China, especially in the cotton field soils with long-term plastic film mulching in Xinjiang, making them one of the primary sources of environmental pollution in this region (Xu Y. et al., 2022). Among them, DBP is one of the most widely used PAEs, and studies have shown that it can enter organisms through the food chain, causing various toxic effects (Zhao et al., 2019; Paluselli et al., 2019). Given the widespread distribution of PAEs in the environment and their adverse impacts on human and environmental health, effective strategies for PAEs pollution remediation are needed. Bioremediation is considered an environmentally friendly and cost-effective method for restoring contaminated ecosystems. In this study, several bacterial strains capable of degrading DBP were isolated from the long-term plastic film mulched cotton field soil in Xinjiang, among which *P. megaterium* P-7 exhibited unique degradation characteristics. This strain demonstrated the ability to adapt to high concentrations of DBP and utilize it as a carbon source for growth, with a significantly shorter degradation time compared to other DBP degrading strains. Some studies reported that degrading strains such as *Paracoccus kondratievae* BJQ0001 (Xu et al., 2020), *Mycobacterium* sp. YC-RL4 (Ren et al., 2016), *G. alkanivorans* YC-RL2 (Nahurira et al., 2017), generally degraded DBP within 120 h, 5–7 days, or even several months. Notably, this study also found that *P. megaterium* P-7 showed strong stress tolerance in extreme environments. These findings indicate that *P. megaterium* P-7 has strong survival ability in harsh environments, making it highly adaptable to various complex ecological environments. Therefore, it shows significant potential and advantages in removing DBP from contaminated environment.

Environmental factors play a significant role in the biodegradation of PAEs. The high biodegradation rate of *P. megaterium* P-7 under neutral and alkaline conditions would be beneficial for removing DBP in such environment, which was similar to the biodegradation of DEP by *Comamonas* sp. USTBZA1 (Zhao et al., 2023). This might be attributed to the enhanced enzyme stability and increased substrate accessibility under optimal pH condition. Additionally, the presence of metal ions in the environment had been shown to promote the degradation of DBP. Even low concentrations of Fe^2+^ could significantly enhance the DBP degradation efficiency of *P. megaterium* P-7. This result might be due to Fe^2+^ acting as a cofactor of enzymes, significantly enhancing the activity of DBP degrading enzymes, such as UbiD-family decarboxylases (Mergelsberg et al., 2017). Moreover, the growth and proliferation of the strain, as well as its ability to degrade toxic pollutants, are strongly influenced by several other factors, such as nutritional requirements, cellular energy status, and the physicochemical cultivation conditions (Feng L. et al., 2018; Feng N. et al., 2018). Therefore, it is crucial to obtain optimal conditions for efficient and low-cost degradation of DBP. In this study, the optimal conditions obtained were successfully applied to achieve the degradation of various PAEs (DMP, DEP, DBP, BBP, and DEHP) by *P. megaterium* P-7, demonstrating a broad range of substrates utilization compared to other strains. In comparison, *P. megaterium* P-7 exhibited better degradation performance for short-chain alkyl PAEs than for long-chain alkyl PAEs, particularly demonstrating a substrate preference for DBP and DEP, as the stereospecific blockade of long-alkyl chains might prevent hydrolases from binding to PAEs (Boll et al., 2020). In summary, the optimal conditions obtained in this study provide a reliable reference for the effective and rational utilization of this train in the bioremediation of PAE contaminated environments.

In typical PAEs degradation, the known microbial degradation pathway begins with the hydrolysis of the alkyl side chain, and further steps are accomplished by decarboxylases, dioxygenases, and the following dehydrogenases (Xu et al., 2020). Initially, PAEs are converted into PA through direct hydrolysis of ester bonds. PA can then be transformed into BA through decarboxylation reaction, which is commonly considered a metabolic step typically associated with anaerobic microorganisms (Xu et al., 2021). Following this, the benzene ring undergoes cleavage, facilitating subsequent metabolic reactions (Xu et al., 2021). This work speculated the possible metabolic mechanism and pathway of DBP in *P. megaterium* P-7 through whole-genome sequencing and metabolomics. The esterases, lipases, and hydrolases were likely responsible for the hydrolysis of PAEs, which was a key step in the degradation process (Xu et al., 2021). Their identification helps to reveal the genetic basis of *P. megaterium* P-7 for PAEs degradation and elucidate the molecular degradation mechanisms. PA was a central metabolite formed during the hydrolysis of DBP and is derived from the primary metabolite MBP. Specific genes involved in the degradation of PA have been found in some Gram-negative bacteria, such as the phthalate dioxygenase gene cluster involved in the conversion of PA to PCA (*oph* and *pht* gene cluster) (Zhao et al., 2023; Feng L. et al., 2018), as well as decarboxylase genes *pdc* and *bsdCD* involved in the conversion of PA to BA. However, this study did not identify phthalate dioxygenase genes but instead found an aromatic ring-hydroxylating dioxygenase (gene0497, gene3652, and gene5204) with similar function. Furthermore, BA and PCA were converted into catechol by *P. megaterium* P-7, which was the key intermediate before the ring cleavage of aromatic compounds (Song et al., 2022). Further combined with the identification of intermediate products in metabolomics, we proposed three potential pathways for DBP degradation: (1) DBP → MBP → PA → PCA → Catechol, (2) DBP → MBP → BB → BA → Catechol, and (3) DBP → DEP → MEP → PA → PCA → Catechol. Ultimately, the cleavage of catechol ring was achieved through ortho-or meta-degradation pathways catalyzed by catechol 1,2-dioxygenase and catechol 2,3-dioxygenase, with the final metabolic intermediates entering the TCA cycle for cell growth (Al-Khalid and Ei-Naas, 2012).

## 5 Conclusion

This study isolated a highly efficient DBP degrading strain, *P. megaterium* P-7, from the soil of long-term mulching cotton fields in Xinjiang. Under optimized conditions, it completely degraded 100 mg/L DBP within 20 h, demonstrating superior adaptability to neutral/alkaline pH and high salinity. In addition, by optimizing cultivation conditions, the degradation efficiency was significantly improved, providing a solid theoretical foundation for the large-scale application of bioremediation technology. Moreover, this strain also exhibited the ability to degrade various PAEs, with a particular preference for short chain PAEs. Genomic and metabolomic analyses revealed that key enzymes such as esterase, hydroxylase, and dioxygenase played critical roles in DBP degradation through sequential hydrolysis, decarboxylation, and aromatic ring cleavage, converting it into harmless metabolites.

Although this work indicates that *P. megaterium* P-7 has significant degradation ability toward DBP and other phthalate esters under laboratory conditions, its practical application in real-world environments remains a crucial next step. Future work should prioritize: (1) in-situ trials to assess degradation efficiency, microbial survival, and adaptability in contaminated soils; (2) ecological impact studies on native microbial communities and soil biodiversity; (3) development of bio-augmentation/bio-stimulation strategies for diverse soil conditions; (4) long-term monitoring of metabolic activity and degradation persistence; and (5) exploration of its ability to degrade other persistent PAEs. Addressing these directions will advance scalable bioremediation solutions for PAE-contaminated agricultural ecosystems, leveraging the enzyme versatility and environmental resilience of *P. megaterium* P-7 to mitigate plasticizer pollution.

## Data Availability

The data that support the findings of this study have been deposited into the Sequence Read Archive of the National Center for Biotechnology information (SRA, NCBI, www.ncbi.nlm.nih.gov/sra) under the BioProject ID PRJNA1250115.

## Footnotes

^1^ http://www.majorbio.com

^2^ http://ccb.jhu.edu/software/glimmer/index.shtml
