# Supplementary material for: Screening, identification, metabolic pathway of di-n-butyl phthalate degrading Priestia megaterium P-7 isolated from long-term film mulched cotton field soil in Xinjiang
Source: Front Microbiol. 2025 Apr 30;16:1538746. doi: 10.3389/fmicb.2025.1538746 (PMC12075219; doi:10.3389/fmicb.2025.1538746)
Supplement: Supplementary file 1 [file Data_Sheet_1.docx]

**Supplementary Materials**

**Screening, identification, metabolic pathway of di-n-butyl phthalate degrading *Priestia megaterium* P-7 isolated from long-term film mulched cotton field soil in Xinjiang**

Yuanyang Yi^1^, Yuxian Wang^2^, Wanqin Liu^2^, Jing Zhu^3^, Meiying Gu^3^, Qiong Jia^3^, Xue Li^4^, Munire Mutalifu^4^, Ling Jiang^5^, Wei Zhang^1*^, Zhidong Zhang^1,2,3,4*^

^1^School of Life Sciences, Xinjiang Normal University, Urumqi 830046, China

^2^College of Life Science and Technology, Xinjiang University, Urumqi 830000, China

^3^Xinjiang Key Laboratory of Special Environmental Microbiology, Institute of Applied Microbiology, Xinjiang Academy of Agricultural Science, Urumqi 830091, China

^4^College of Food Science and Pharmaceutical Science, Xinjiang Agricultural University, Urumqi 830052, China

^5^State Key Laboratory of Materials-Oriented Chemical Engineering, College of Food Science and Light Industry, Nanjing Tech University, Nanjing 211816, China

*Corresponding author.

Zhidong Zhang, Email: zhangzheedong@sohu.com

Xinjiang Key Laboratory of Special Environmental Microbiology, Institute of Applied Microbiology, Xinjiang Academy of Agricultural Science, Urumqi 830091, China

Wei Zhang, Email: zw0991@sohu.com

School of Life Sciences，Xinjiang Normal University，Urumqi 830046, China

**Figure S1** Bacterial growth and DBP degradation in MSM (pH 7.0) in the presence of sucrose (0.19 g/L), beef extract (9.92 g/L), and Fe^2+^ (0.12 g/L) incubated at 30 ℃, 160 rpm for 2 days. Error bars represent the standard deviation of the means of three replicates.


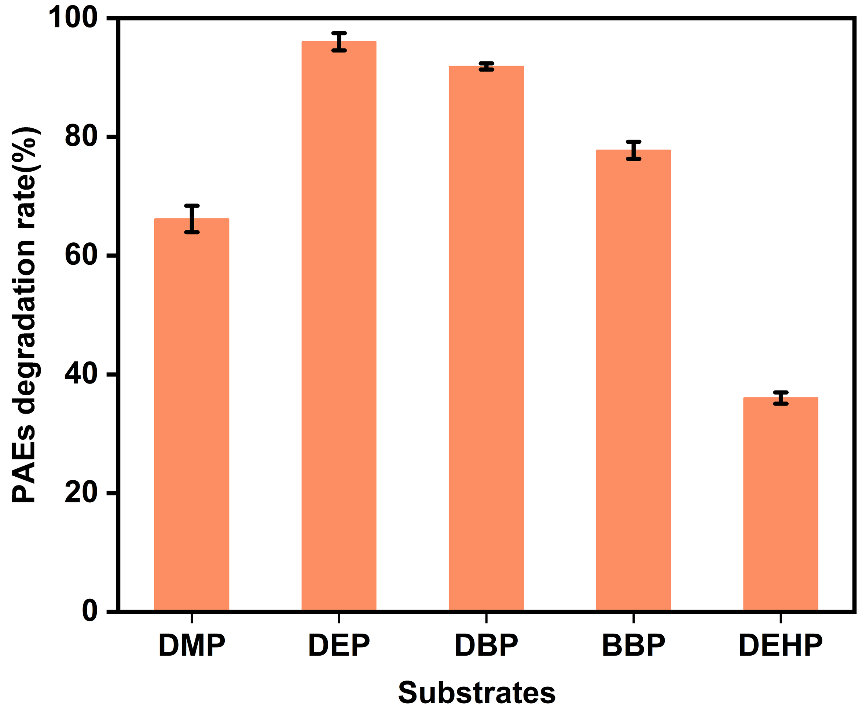


**Figure S2** The degradation rates of different substrates by *P.* *megaterium* P-7.


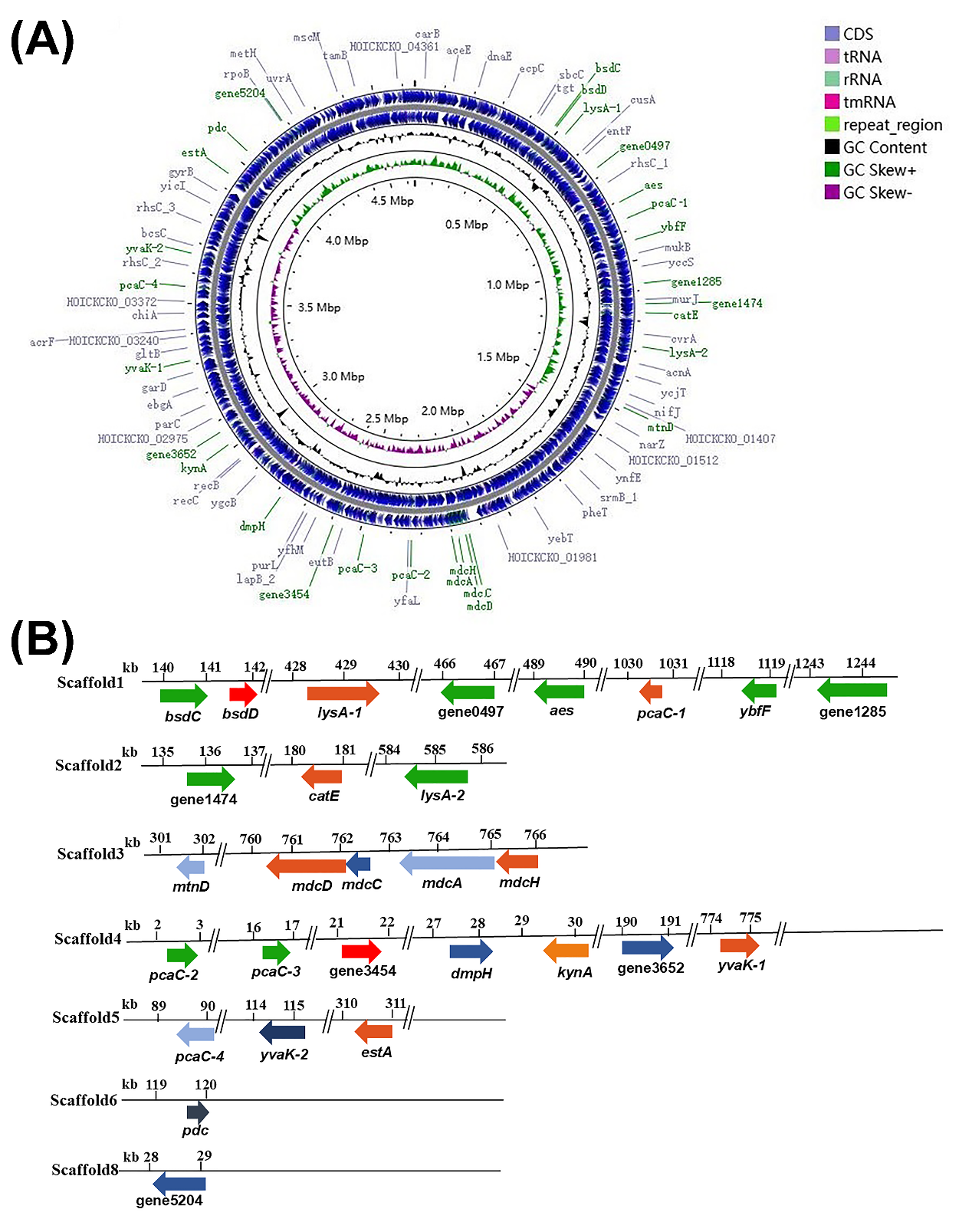


**Figure S3** Key genes arrangement of DBP degradation in *P. megaterium* P-7. (A) Circular genome map of *P. megaterium* P-7 chromosome. The key DBP-degrading genes were labeled by green line. (B) Key genes associated with DBP degradation in *P. megaterium* P-7. The length and direction of the arrow on the map represent the length and coding direction of the genes.


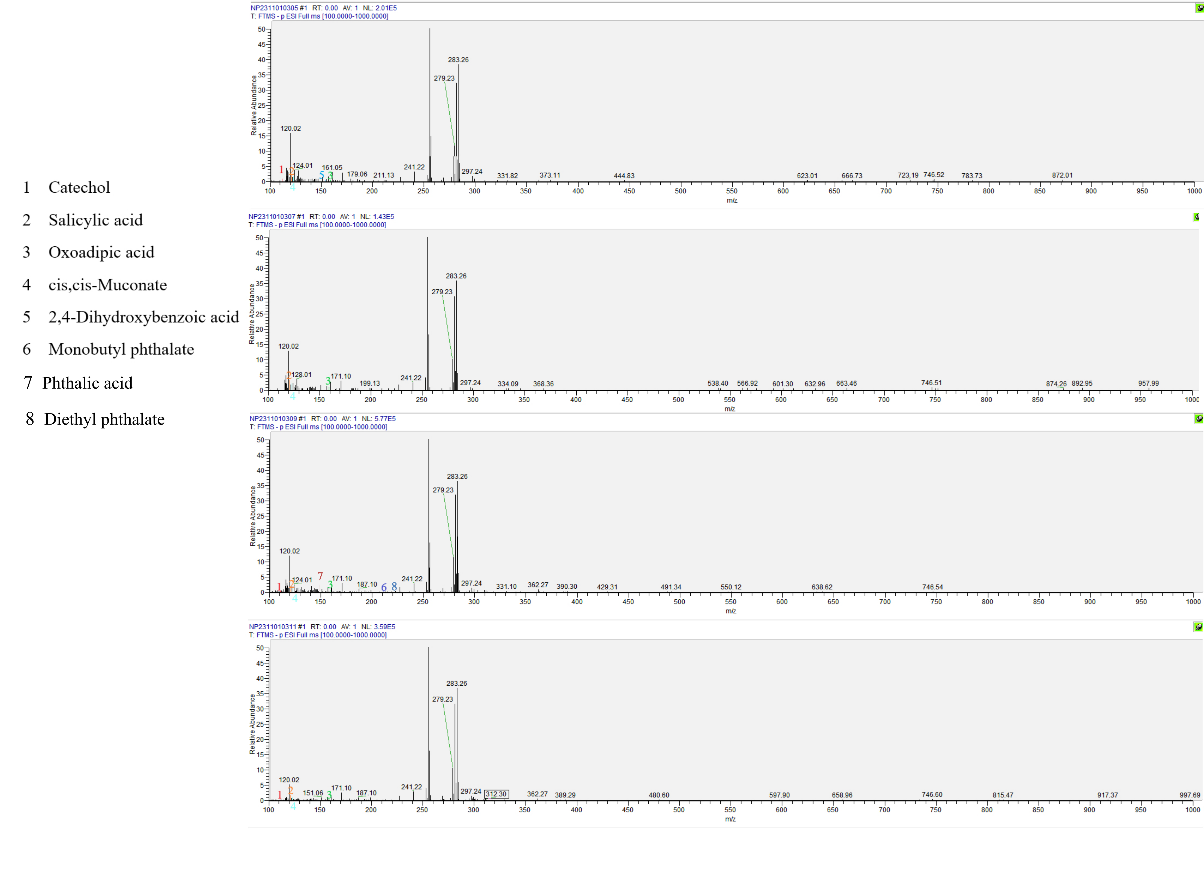


**Figure S4** UHPLC-MS/MS of metabolites from DBP degradation identified in *P. megaterium* P-7.

**Table S1** Comprehensive score and ranking of the effect of single factor on DBP degradation of strain *P. megaterium* P-7.

| Item | y1 | y2 | y | \| y \| | Rank |
| --- | --- | --- | --- | --- | --- |
| X1 | -0.61 | 0.74 | -0.25 | 0.25 | 5 |
| X2 | -3.35 | -0.97 | -2.62 | 2.62 | 1 |
| X3 | 1.07 | 0.73 | 0.94 | 0.94 | 4 |
| X4 | 1.51 | -0.33 | 0.99 | 0.99 | 3 |
| X5 | 1.65 | -0.51 | 1.05 | 1.05 | 2 |

Note: X1 refers to pH; X2 refers to metal ions; X3 refers to inoculation amount; X4 refers to carbon source；X5 refers to nitrogen source.

**Table S2** The symbols and levels of three independent variables used in Box-Behnken design.

| **Independent variables** | **Symbol** | **Code levels of variables** | | |
| --- | --- | --- | --- | --- |
|  |  | -1 | 0 | 1 |
| Sucrose (g/L) | *X*_1_ | 0.15 | 0.2 | 0.25 |
| Beef extract (g/L) | *X*_2_ | 6 | 8 | 10 |
| Fe²⁺ (g/L) | *X*_3_ | 0.1125 | 0.15 | 0.1875 |

**Table S3** The 16S rRNA gene sequence alignment results of DBP degradation strains.

| No. | Strain | Identification (nearest type strain) | Genebank number | Similarity (%) |
| --- | --- | --- | --- | --- |
| 1 | L-2 | *Dietzia cinnamea* strain IMMIB RIV-399 | NR042390 | 99.10 |
| 2 | L-7 | *Priestia flexa* strain NBRC 15715 | NR113800 | 99.00 |
| 3 | L-15 | *Microbacterium paraoxydans* strain JN-1 | KP064030 | 99.50 |
| 4 | P-7 | *Priestia* *megaterium* NBRC 15308 | JMH01000057 | 99.00 |
| 5 | P-9 | *Microbacterium resistens* strain DMMZ 1710 | NR_026437 | 99.00 |
| 6 | P-14 | *Priestia megaterium* strain IMA 13418 | MK424276 | 100.00 |
| 7 | P-16 | *Microbacterium algeriense* strain G1 | MK480726 | 100.00 |
| 8 | P-21 | Sphingobacterium changzhouense strain N7 | NR135709 | 100.00 |

**Table S4** The physiological and biochemical characteristics of *P. megaterium* P-7.

| Physiological and biochemical test | Results | Physiological and biochemical test | Results |
| --- | --- | --- | --- |
| Gram staining | + | Dissolved inorganic phosphorus | + |
| NaCl (10%) tolerance | + | Potassium solubilizing | - |
| Cold (4℃) tolerance | + | Indol reaction | + |
| Thermotolerance (45℃) | + | ACC deaminase | - |
| Alkali tolerance (pH =11) | + | Catalase production | + |
| Phenol tolerance (0.1%) | w | Starch hydrolysis | + |
| Siderophore-producing | + | Oxidase production | - |
| Dissolved organic phosphorus | - | Protease production | + |

Note: +: Positive; -: Negative.; w: weak.

**Table S5** Comparison of DBP biodegradation by different bacterial strains.

| **Species** | **Isolation** | **Degradation rate (mg/L)** | **References** |
| --- | --- | --- | --- |
| *P. megaterium* P-7 | Cotton field | 100%, 20h, 100mg/L | This work |
| *Bacillus megaterium* YJB3 | Canna indica root tissue | 82.5%, 5 days, 100mg/L | 1 |
| *Streptomyces* sp. FZ201 | Root surface of wild rice | 98%, 144h, 800 mg/L | 2 |
| *Pseudomonas* sp. YJB6 | Soil | 95.2%, 5d, 500 mg/L | 3 |
| *Pseudomonas sp.DNB-S1* | Soil | 90%, 2d, 500 mg/L | 4 |

**Table S6** Genomic characterization and gene annotation of *P. megaterium* P-7.

| **Features** | **Value** |
| --- | --- |
| **Assembly statistics** |  |
| Genome size (bp) | 5,567,352 |
| G+C content (%) | 37.61 |
| Total scaffolds number | 55 |
| Scaffold N50 (bp) | 1,008,390 |
| Scaffold N90 (bp) | 105,962 |
| Total Contigs number | 92 |
| Contig N50 | 901,641 |
| Contig N90 | 62,227 |
| **Gene statistics** |  |
| Gene number | 5,773 |
| Protein-coding genes | 5,653 |
| GC content in gene region (%) | 38.64 |
| rRNA genes | 103 |
| tRNA genes | 17 (1 16S rRNA, 1 23S rRNA, 15 5S rRNA) |
| **Annotation statistics** |  |
| Coding gene assigned to GO | 3,230 |
| Coding gene assigned to COG | 4,150 |
| Coding gene assigned to KEGG | 2,928 |
| Coding gene assigned to NR | 5,697 |
| Coding gene assigned to Swiss-Prot | 4,264 |

**Table S7** Prediction of gene promoter sequences using the PromPredict software.

| **Gene ID** | **Location** | **Upstream Pos** | **Promoter Len (bp)** | **Promoter Seq** |
| --- | --- | --- | --- | --- |
| gene0529 | Scaffold1 | 142 | 96 | aatatcatcctctttatttaattgttatacccatttttaatcaaaacaaacaaaagtctgtcgagaagacaatatattggattttttctgaaattt |
| gene1165 | Scaffold1 | 135 | 147 | ggatacataaaataatctaatatctttcctaataagataagaggtgaaggaaatcttcattccttcgctaatatatagatgaaccttaataaactagattttaattggtgaaatctgagttaggagttgatctaatttgaaaatgcg |
| gene1474 | Scaffold2 | 142 | 134 | attctctaatttacgtcttctgaaattcgcactttctacaaagttccatatttcatgttagaactccttacataaattgtgtgaattttctttttttatattacccttagtaaaagagcttacaaaagaaggga |
| gene1517 | Scaffold2 | 103 | 127 | atgatagtaaaataaataacttgacgaaataaagttataaaaatataataagttacataaagtaagttgataaaaaaatgatatattcaaaaggagaaataatcatgaactttcaccgtgcacctca |
| gene3652 | Scaffold4 | 127 | 144 | caacattttcttgttattgacaaacaggctgtatatcctttaaaattatctttaattaaagataattaaattaaagataattttagtagaagttaaaaatttatatattatgaaaagaggcggagcagatggaattgttagggt |

**Table S8** UHPLC-MS/MS of metabolites from DBP degradation identified in *P. megaterium* P-7.

| **No.** | **ID** | **name** | **mz** | **rt** | **exact_mass** | **formula** | **KEGG_Pathway** |
| --- | --- | --- | --- | --- | --- | --- | --- |
| 1 | M111T578 | Catechol | 111.0921 | 578.3 | 110.0368 | C_6_H_6_O_2_ | Xenobiotics biodegradation and metabolism; Global and overview maps |
| 2 | M121T551 | Salicylic acid | 121.0287 | 551.5 | 138.0317 | C_7_H_6_O_3_ | Signal transduction; Digestive system; Xenobiotics biodegradation and metabolism; Metabolism of terpenoids and polyketides; Amino acid metabolism; Chemical structure transformation maps |
| 3 | M159T567 | Oxoadipic acid | 159.0299 | 567.1 | 160.0372 | C_6_H_8_O_5_ | Energy metabolism; Amino acid metabolism; Global and overview maps |
| 4 | M123T84 | cis,cis-Muconate | 123.0084 | 83.7 | 142.0266 | C_6_H_6_O_4_ | / |
| 5 | M153T567 | 2,4-Dihydroxybenzoic acid | 153.0185 | 567.4 | 154.0266087 | C_7_H_6_O_4_ | / |
| 6 | M221T314 | Monobutyl phthalate | 221.0805 | 313.7 | 222.0892089 | C_12_H_14_O_4_ | / |
| 7 | M149T329 | Phthalic acid | 149.0228 | 329.2 | 166.0266 | C_8_H_6_O_4_ | Membrane transport; Xenobiotics biodegradation and metabolism; Global and overview maps |
| 8 | M223T550 | Diethyl phthalate | 223.0973 | 550 | 222.0892 | C_12_H_14_O_4_ | / |

**References**

[1] Bai L, Zhang J, Wang Y. Biodegradation of di-n-butyl phthalate (DBP) by a novel endophytic Bacillus megaterium strain YJB3[J]. Environmental Science and Pollution Research, 2021, 28(2): 1925-1933. DOI: 10.1007/s11356-020-10711-9.

[2] Wang Y, Zhang Z, Liu X, et al. Biodegradation of phthalate acid esters and whole-genome analysis of a novel Streptomyces sp. FZ201 isolated from natural habitats[J]. Environmental Pollution, 2020, 259: 113925. DOI: 10.1016/j.envpol.2019.113925.

[3] Zhang L, Li H, Zhao X, et al. Complete biodegradation of di-n-butyl phthalate (DBP) by a novel Pseudomonas sp. YJB6[J]. Environmental Pollution, 2021, 268: 115664. DOI: 10.1016/j.envpol.2020.115664.

[4] Li X, Zhang Y, Wang Z, et al. Complete metabolic study by dibutyl phthalate degrading Pseudomonas sp. DNB-S1[J]. Environmental Science and Pollution Research, 2021, 28(12): 14567-14577. DOI: 10.1007/s11356-021-12795-4.
